# Chronic TLR7 activation promotes expansion of activated CD11c^+^ double-negative B cells and autoreactive humoral responses in lupus-like autoimmunity

**DOI:** 10.3389/fimmu.2026.1920764

**Published:** 2026-08-12

**Authors:** Fernando N. Ferreyra, Laura Almada, Maria S. Martinez, Yair A. Chocobar, Rubén D. Motrich, Juan Pablo Mackern-Oberti, Adriana Gruppi, Virginia E. Rivero

**Affiliations:** 1 Federation of Clinical Immunology Societies (FOCIS) Center of Excellence, Centro de Inmunología Clínica de Córdoba (CICC), Córdoba, Argentina; 2 Centro de Investigaciones en Bioquímica Clínica e Inmunología (CIBICI)-Consejo Nacional de Investigaciones Científicas y Técnicas (CONICET), Facultad de Ciencias Químicas, Universidad Nacional de Córdoba, Córdoba, Argentina; 3 Instituto de Medicina y Biología Experimental de Cuyo (IMBECU), Centro Científico Tecnológico (CCT) Consejo Nacional de Investigaciones Científicas y Técnicas (CONICET), Mendoza, Argentina; 4 Universidad Nacional de Cuyo, Facultad de Ciencias Médicas, Mendoza, Argentina

**Keywords:** ABC-like, ADORA2A, autoantibodies, DN CD11c^+^ B cells, extrafollicular response, imiquimod, lupus, TLR7

## Abstract

Age-associated B cells (ABCs) have emerged as a distinct B cell population associated with aging, chronic inflammation, and autoimmunity. Notably, the expansion of ABC-like CD11c-expressing B cells has been observed in patients with systemic lupus erythematosus and in experimental models of lupus-like disease, suggesting their potential involvement in disease pathogenesis. Although their relevance in autoimmunity is increasingly appreciated, the identity and functional contribution of CD11c^+^ B cell populations arising during chronic Toll-like receptor 7 (TLR7)-driven immune activation remain incompletely defined. In this study, we investigated the role of CD11c^+^CD21^–^CD23^–^ B cells (hereafter DN CD11c^+^ B cells) in a lupus-like model induced by chronic activation of TLR7. Young female C57BL/6 mice were treated with the TLR7 agonist imiquimod for 10 weeks, leading to systemic immune activation characterized by splenomegaly, elevated proinflammatory cytokines, anti-nuclear autoantibodies, and early renal alterations. This was accompanied by a marked remodeling of the B cell compartment, including expansion of non-canonical subsets, particularly DN CD11c^+^ B cells. Phenotypic analysis revealed that DN CD11c^+^ B cells exhibited an activated profile, with increased expression of T-bet, TLR7, costimulatory molecules, and activation-associated markers, as well as a higher proportion of class-switched cells. Functional assays demonstrated that DN CD11c^+^ B cells from imiquimod-treated mice were hyperresponsive to TLR7 stimulation. Furthermore, DN CD11c^+^ B cells were a major ex vivo source of autoreactive antibodies, including IgG directed against nuclear antigens, whereas conventional B cell subsets showed minimal contribution. In addition, DN CD11c^+^ B cells were detected in renal tissue prior to the development of overt pathology, suggesting a role in early tissue infiltration. Importantly, pharmacological activation of the adenosine A2A receptor preferentially reduced the frequency of DN CD11c^+^ B cells, autoantibody production, and immune cell infiltration, while largely sparing conventional B cell subsets. Collectively, these findings identify expanded and activated DN CD11c^+^ B cells as important effectors linking chronic innate immune activation to autoreactive humoral responses and highlight A2A receptor signaling as a potential strategy to selectively modulate pathogenic B cell responses in lupus-like autoimmunity.

## 1 Introduction

Systemic lupus erythematosus (SLE) is a chronic, multiorgan autoimmune disease characterized by loss of immune tolerance, sustained immune activation, and the production of autoantibodies against nuclear antigens (1–3). These autoantibodies drive immune complex formation and deposition, chronic inflammation, and tissue damage, particularly in the kidney (1–3). Although substantial progress has been made in understanding SLE pathogenesis, the mechanisms that initiate and sustain nuclear antigen-specific autoreactive B cell responses remain incompletely understood (1–4).

B cells contribute to SLE not only through antibody production but also via antigen presentation and cytokine secretion, thereby shaping T cell responses and amplifying inflammation (5, 6). The functional heterogeneity of B cells in disease and the development of B cell-targeted therapeutic strategies have been reviewed recently (7). In this context, non-canonical B cell subsets that emerge under chronic inflammatory conditions have gained increasing attention. Among these, a population of CD11c^+^CD21^–^CD23^–^ B cells (hereafter DN CD11c^+^ B cells), commonly associated with age-associated B cells (ABCs) and characterized by low levels of CD21, CD11c positivity, and expression of the transcription factor T-bet, has been identified as a distinct population that expands during aging, chronic infections, and autoimmune diseases (5, 8–10). Throughout this study we use “DN” in the murine sense, to denote CD21−CD23− B cells further resolved by CD11c expression, which differs from the human IgD−CD27− double-negative nomenclature. We refer to this CD21−CD23−CD11c+ population as DN CD11c+ (ABC-like) B cells. DN CD11c^+^ B cells are enriched for autoreactive specificities and exhibit enhanced responsiveness to innate stimuli, increased antigen-presenting capacity, and proinflammatory cytokine production (11–15). Importantly, these cells have been proposed as precursors of autoreactive plasmablasts and plasma cells, suggesting a direct contribution to pathogenic humoral responses (16–19).

Innate immune signaling, particularly through Toll-like receptor 7 (TLR7), plays a critical role in the activation and differentiation of ABCs. TLR7 recognizes single-stranded RNA and endogenous nucleic acids released during cellular damage, thereby linking tissue stress to immune cell activation (20–22). Activation of TLR7 promotes B cell activation, type I interferon (IFN-I) production, and amplification of autoreactive immune responses. Dysregulated TLR7 signaling has been strongly associated with lupus pathogenesis in both murine models and human disease (23–25). In this regard, experimental models based on TLR7 stimulation, such as repeated administration of the TLR7 agonist imiquimod, reproduce key features of lupus-like disease, including splenomegaly, systemic inflammation, autoantibody production, and renal alterations (26, 27). Importantly, TLR7 escapes X chromosome inactivation in immune cells, contributing to enhanced responsiveness in females and potentially amplifying nucleic acid–driven immune activation (28–30). Female-biased lupus susceptibility has also been linked to interactions between TLR7 signaling, X chromosome dosage, and sex hormone pathways, including crosstalk between estrogen receptor signaling and type I interferon responses (28–30).

DN CD11c^+^ B cells are highly responsive to TLR7-mediated signals and include subsets that can arise through extrafollicular differentiation pathways (31–33). Unlike germinal center responses, extrafollicular pathways enable rapid generation of antibody-secreting cells under inflammatory conditions but operate with fewer tolerance checkpoints, potentially favoring autoreactivity (34–36). In spontaneous lupus models, ABCs display features consistent with pathogenic effector cells, including oligoclonal expansion and enrichment in interferon-stimulated gene signatures (37–41). Moreover, factors such as TLR7 gene dosage and signaling adaptors, including BANK1, have been shown to regulate ABC expansion and function (41–45). Several questions nevertheless remain open. The developmental origin of ABCs, germinal center versus extrafollicular, is still debated, and their relationship to the DN2 and atypical-memory populations is incompletely resolved. In addition, the imiquimod model is widely used as an inducible model of TLR7-driven lupus-like disease (26). Although CD11c+ B cells are known to expand following TLR7 stimulation (11) and to contribute to lupus pathogenesis (32), the phenotype, functional properties, tissue distribution and pharmacological modulation of these cells have not been characterized in an integrated way within this model. Here we investigate which B cell subset has the capacity to generate autoreactive humoral responses in chronic TLR7-driven disease, how sustained TLR7 exposure shapes it functionally, when these cells reach the kidney, and whether they can be selectively targeted.

In this study, we investigated the impact of chronic TLR7 activation on the expansion and functional programming of DN CD11c^+^ B cells (the ABC-like population in our model). To this end, we employed a lupus-like model induced by repeated topical administration of the TLR7 agonist imiquimod in young female C57BL/6 mice. We evaluated systemic immune activation, remodeling of the B cell compartment, and the phenotype and function of DN CD11c^+^ B cells, as well as their contribution to autoantibody production and tissue infiltration. In addition, we assessed the therapeutic potential of targeting DN CD11c^+^ B cells through pharmacological activation of the adenosine A2A receptor. Our findings delineate a mechanistic link between sustained innate immune activation and the emergence of pathogenic DN CD11c^+^ B cell responses, and support the targeting of the A2A receptor as a strategy to modulate autoreactive B cell immunity.

## 2 Materials and methods

### 2.1 Mice

All animal procedures were approved by and conducted in accordance with the Institutional Animal Care and Use Committee of the Facultad de Ciencias Químicas, Universidad Nacional de Córdoba (Ordinance OHCD-2021-5-E-UNC-DEC#FCQ). Female C57BL/6 wild-type mice (WT; JAX:000664), 7–8 weeks old at the start of the experiment, were bred and maintained under specific pathogen-free (SPF) conditions at the Animal Facility of CIBICI-CONICET, Facultad de Ciencias Químicas, Universidad Nacional de Córdoba. Mice were housed with controlled temperature and humidity, a 12-h light/dark cycle, and ad libitum access to food and water. At the end of the 10-week protocol, after induction of anesthesia by isoflurane inhalation (2%), euthanasia was carried out via cervical dislocation by trained personnel, in accordance with institutional guidelines and the AVMA Guidelines for the Euthanasia of Animals (2020).

### 2.2 TLR7 lupus model and A2A activation

A chronic lupus-like condition was induced through topical activation of TLR7. The skin of the right ear was treated with 1.5 mg of Miquimod cream (Imiquimod 5%), administered three times per week for 10 weeks. Control mice received identical handling and were treated on the same schedule with glycerin instead of Miquimod. This strategy is conceptually based on previously established TLR7-driven lupus-like models, in which topical TLR7 agonism has been shown to induce systemic autoimmunity in multiple wild-type mouse strains (C57BL/6, BALB/c, and FVB/N) (26).

A subset of IMQ-treated mice additionally received CGS-21680, an adenosine A2A receptor agonist. CGS-21680 (Abcam Biochemicals, Lot APN13052-1-1) was administered intraperitoneally at 2.5 mg/kg, twice per week, starting at week 4 and continuing until the end of the 10-week protocol. The compound was prepared following the manufacturer’s recommendations and diluted in 200 *μ*L PBS. The experimental timing and group allocation were based conceptually on previous A2A receptor activation studies (46). Control mice received equivalent injections of vehicle.

All experiments were independently reproduced using at least two distinct biological cohorts.

### 2.3 Collection of tissue and blood

Following euthanasia, spleens were harvested and single-cell suspensions were obtained by mechanical dissociation. Two parallel processing protocols were used: for flow cytometry and cell sorting, RBCs were depleted by ACK lysis buffer, whereas for ex vivo culture experiments, mononuclear cells were isolated by Ficoll-Hypaque density gradient (Sigma, CAT#10831).

For longitudinal serological analyses, peripheral blood was collected at weeks 0, 4, 6, and 8 by submandibular (facial vein) puncture in conscious, manually restrained mice. At the experimental endpoint (week 10), blood was obtained by cardiac puncture following euthanasia. In all cases, blood was centrifuged at 500 × g for 10 min; the serum was collected and stored at −20 °C for further analysis, and the cell pellet from terminal samples was subjected to lysis buffer treatment to isolate leukocytes, which were subsequently analyzed by flow cytometry.

### 2.4 Kidney collection and processing for flow cytometry

Kidneys were collected immediately after euthanasia. Mice were perfused via cardiac puncture with cold PBS until the effluent cleared. Both kidneys were excised; one kidney was processed for flow cytometry, while the contralateral kidney was reserved for histological analysis.

For flow cytometry, kidney tissue was minced and mechanically dissociated by gently pressing the tissue through a 70-*μ*m mesh using the plunger of a syringe. The resulting cell suspension was washed with PBS + 2% FBS, subjected to erythrocyte lysis with ACK buffer, washed again, and filtered through a 70-*μ*m strainer to remove debris and aggregates. Total cell numbers and initial viability were assessed by Trypan Blue exclusion (1:1 dilution) using a Neubauer chamber. Prior to surface staining, cell viability was evaluated using LIVE/DEAD Fixable Aqua (Invitrogen, CAT# L34966) as part of the flow cytometry panel. All samples corresponded to female mice and were processed under identical conditions.

### 2.5 Histological processing of kidney tissue

The contralateral kidney from each mouse was collected immediately after euthanasia. Kidneys were longitudinally bisected along the major axis and immersed in 10% neutral-buffered formalin (NBF). After fixation, all subsequent histological processing was performed by the Histotechnology Facility of CIBICI-CONICET following standardized protocols. This included dehydration, paraffin embedding, sectioning (4–5 *μ*m), and hematoxylin and eosin (H&E) staining. Staining quality and tissue integrity were verified by the facility prior to imaging.

### 2.6 Serum biochemistry and cytokine analysis

Serum urea and creatinine concentrations were measured using colorimetric enzymatic assays, performed strictly according to the manufacturer’s instructions. Urea was quantified using the UREA COLOR 2R kit (Wiener Lab, Cat. 1810050), and creatinine was measured using the Creatinina Cinética AA kit (Wiener Lab, Cat. 1009254). Circulating cytokines were quantified in serum using a bead-based multiplex assay (LEGENDplex Mouse Inflammation Panel, BioLegend, CAT# 740446) according to the manufacturer’s instructions; samples were acquired by flow cytometry and analyzed with the LEGENDplex Data Analysis Software.

### 2.7 Detection of autoantibodies by indirect immunofluorescence

Antinuclear antibodies (ANA) were assessed using HEp-2 cell substrate slides (Biocientífica, Cat. NT00-12), and anti–double-stranded DNA (anti-dsDNA) antibodies were evaluated using *Crithidia luciliae* substrate slides (Biocientífica, Cat. NT11-7). Serum samples were diluted 1:5 in PBS and processed according to the manufacturer’s instructions. Immunofluorescence imaging was performed using a Leica DMI8 fluorescence microscope under identical acquisition settings for all samples.

### 2.8 Quantification of specific autoantibodies by ELISA

For the detection of specific autoantibodies, 96-well plates were coated with 5 *μ*g/mL of calf thymus double-stranded DNA (D3664-5×2MG, Sigma-Aldrich) or 5 *μ*g/mL of histones (ATH01-02, Arotec Diagnostic Limited), diluted in carbonate buffer (pH 9.5), and incubated overnight at 4 °C. Then, plates were washed three times with PBS containing 0.05% Tween-20 (PBS-T) and blocked with PBS containing 3% BSA for 2 hours at 37 °C. Serum samples were diluted 1:5000 and culture supernatants 1:2 in PBS containing 1% BSA, and incubated overnight at 4 °C; all samples were assayed in technical duplicates. Specific autoantibodies were detected using HRP-conjugated goat anti-mouse antibodies (IgG, IgG1, IgG2b, IgG2c, IgG3, or IgM; SouthernBiotech), incubated for 1 hour at 37 °C. Peroxidase activity was developed using TMB substrate (BD Biosciences). The reaction was stopped with 1 N H_2_SO_4_, and absorbance was measured at 450 nm using a microplate reader (Bio-Rad 450). Results were expressed as absorbance values (450 nm).

### 2.9 Culture and stimulation of B cells

Splenic mononuclear cells (1 × 10^6^ cells/well) from untreated and IMQ-treated mice were cultured in RPMI 1640 supplemented with 5% heat-inactivated FBS, 2 mM L-glutamine, 50 *μ*M 2-mercaptoethanol, 100 U/mL penicillin, and 100 *μ*g/mL streptomycin. Cells were stimulated with goat F(ab’)_2_ anti-mouse IgM (4 *μ*g/mL; SouthernBiotech, Cat. 1023-01) plus goat F(ab’)_2_ anti-mouse IgG (4 *μ*g/mL; SouthernBiotech, Cat. 1033-01), alone or combined with R848 (0.625 *μ*g/mL; InvivoGen), in 96-well U-bottom plates with 200 *μ*L of medium per well, for 24 h at 37 °C in a humidified incubator with 5% CO_2_, adapted from (47). The phenotypic response of DN CD11c^+^ B cells was subsequently analyzed by flow cytometry.

For sort-purified cultures, FO, MZ, DN CD11c^–^ and DN CD11c^+^ B cells were FACS-sorted from splenocytes following the gating strategy detailed in the following section (**Supplementary Figure 1C**), and cultured at 2 × 10^5^ cells/well for 72 h under the same medium and stimulation conditions described above. Supernatants were assayed for anti-dsDNA and anti-histone autoantibodies by ELISA.

### 2.10 Flow cytometry and FACS

Flow cytometry was performed using standard procedures. For the identification of different cell populations, the following anti-mouse antibodies were used: B220-PerCP-Cy5.5 (clone RA3–6B2, eBioscience), CD23-PE-Cy7 (clone B3B4, eBioscience), CD21/35-eFluor450 (clone eBio4E3, eBioscience), T-bet-eFluor660 (clone eBio4B10, eBioscience), CD11c-PE-CF594 (clone N418, BD Biosciences), CD11b-APC-eFluor780 (clone M1/70, eBioscience), CD19-PE-Cy7 (clone 1D3, eBioscience), CD138-BV421 (clone 281-2, BD Biosciences), CD73-PE (clone TY/11.8, eBiosciences), PD-L1-BV421 (clone MIH5, BD Biosciences), PD-L2-PE (clone TY25, eBioscience), IgD-BV605 (clone 11-26c.2a, BD Biosciences), IgM-PE (clone R6-60.2, BD Biosciences), MHC-II-AlexaFluor700 (clone M5/114.15.2, BioLegend), CD39-APC-AlexaFluor700 (clone 24DMS1, Invitrogen), TLR7-PE (clone A94B10, BioLegend), Ki67-FITC (clone 11F6, BioLegend), CD80-PE (clone 16-10A1, BioLegend), CD21/35-FITC (clone 7G6, BD Biosciences), CXCR3-PE (clone CXCR3-173, BioLegend), CXCR4-BV605 (clone L276F12, Biolegend), CXCR5-FITC (clone 2G8, BD Biosciences), CD44-AlexaFluor700 (clone IM7, eBioscience), FAS-PE (clone SA367H8, Biolegend), CD3-BV421 (clone 145-2C11, BioLegend), LIVE/DEAD Fixable Aqua (CAT# L34966, Invitrogen), CD8a-BV650 (clone 53-6.7, BioLegend), CD4-PerCP-Cy5.5 (clone RM4-5, eBioscience), FoxP3-AlexaFluor647 (clone MF-14, BioLegend), CD62L-APC-AlexaFluor750 (clone MEL-14, eBioscience), CD69-PE (clone H1.2F3, eBioscience), B220-PE-Cy7 (clone RA3-6B2, eBioscience), CD45-BV785 (clone 30-F11, BioLegend), CD4-AlexaFluor488 (clone GK1.5, eBioscience), IFN-*γ*-PE (clone XMG1.2, eBioscience), IL-17A-PE (clone eBio17B7, eBioscience), and PD1-PE (clone J43, eBioscience).

Cells were incubated with an Fc-blocking antibody (anti-CD16/CD32, clone 2.4G2, BD Biosciences) for 10 minutes at 4 °C. Subsequently, for surface staining, single-cell suspensions were washed with cold FACS buffer (PBS containing 2% FBS) and incubated with fluorochrome-conjugated antibodies for 20 minutes at 4 °C. For intracellular cytokine detection, cells were stimulated for 2 hours with 100 ng/mL phorbol 12-myristate 13-acetate (PMA, Sigma-Aldrich) and 1 *μ*g/mL ionomycin (Sigma-Aldrich), followed by an additional 2 hours in the presence of 5 *μ*g/mL Brefeldin A (GolgiPlug, eBioscience/Thermo Fisher Scientific) to block cytokine secretion. This procedure was used to evaluate intracellular production of IFN-*γ* and IL-17. After surface staining, cells were fixed and permeabilized using the Cytofix/Cytoperm kit (BD Biosciences, CAT# 554714), according to the manufacturer’s instructions. For intracellular staining of Foxp3, MHC-II, Ki67, and T-bet, the Foxp3/Transcription Factor Staining Buffer Set (eBioscience, Thermo Fisher Scientific; CAT# 00-5523-00) was used after surface staining, following the manufacturer’s instructions.

For cell sorting experiments, splenocytes were isolated from pools of 2–3 IMQ-treated young female C57BL/6 mice at the end of the 10-week protocol, age- and treatment-matched to the *in vivo* experiments. Following spleen mechanical dissociation and RBC lysis, cells were stained with fluorochrome-conjugated antibodies and dead cells were excluded using the LIVE/DEAD Fixable Near-IR Dead Cell Stain Kit (Invitrogen, CAT# L10119). Viable cell populations were sorted according to the following gating strategies: FO (B220^+^ CD23^+^ CD21^lo^/–), MZ (B220^+^ CD23^–^ CD21^+^), DN CD11c^–^ B cells (B220^+^ CD23^–^ CD21^–^ CD11c^–^), and DN CD11c^+^ B cells (B220^+^ CD23^–^ CD21^–^ CD11c^+^) (**Supplementary Figure 1C**). Cell sorting was performed using a BD Biosciences FACSAria II.

Flow cytometry data acquisition was performed on a BD LSR Fortessa and a BD FACSCanto II cytometer and analyzed using the FlowJo v10.10 software (TreeStar/FlowJo LLC). Representative gating strategies for T and B cell subpopulations are shown in **Supplementary Figure 1**.

### 2.11 Statistical analysis

Data are presented as mean ± standard error of the mean (s.e.m.). Biological replicates correspond to individual mice; n represents the number of animals per group. Mice were randomly assigned to experimental groups and, when possible, distributed across cages to minimize cage effects.

All statistical analyses were performed on data obtained exclusively from young female mice. Normality and homogeneity of variance were assessed using Shapiro–Wilk and Levene’s tests, respectively. Comparisons between two groups were conducted using unpaired two-tailed Student’s t tests (with Welch’s correction when variances were unequal) or Mann–Whitney tests when data were not normally distributed. For experiments involving more than two groups, one-way ANOVA with Tukey’s post-test (or Kruskal–Wallis with Dunn’s test) was used. Ex vivo stimulation experiments, which followed a two-factor design (*in vivo* treatment and ex vivo R848 stimulation), were analyzed by two-way repeated-measures ANOVA with individual mouse as a blocking factor—to account for the paired stimulated and unstimulated cultures obtained from the same animal—followed by Šidák’s multiple-comparisons post-test. For longitudinal autoantibody measurements, linear mixed-effects models were fitted with treatment, time and their interaction as fixed effects, and individual mouse as a random effect to account for repeated measures within animals; cohort was included as an additional random effect when biological replicates from independent experimental runs were combined, and pairwise comparisons at individual timepoints were derived from estimated marginal means (emmeans). For panels comprising multiple analytes measured in parallel—the serum cytokine panel and the multiparameter flow cytometry phenotyping panels—p-values were adjusted for multiple comparisons within each panel using the Benjamini–Hochberg false discovery rate (FDR) procedure. Analyses were performed in R (v4.5) using the lme4, emmeans and rstatix packages, and in GraphPad Prism (v10.2.3). Statistical significance was defined as p < 0.05; differences with 0.05 < p ≤ 0.07 are referred to as statistical trends.

## 3 Results

### 3.1 Chronic TLR7 agonist treatment induces splenomegaly, systemic inflammation, lymphocyte activation, and autoantibody production

To investigate the impact of chronic TLR7 stimulation on age-associated B cells in autoimmunity, we first established a lupus-like model based on repeated topical administration of imiquimod (IMQ) in young female C57BL/6 mice, and used it as a defined experimental setting in which to dissect the role of DN CD11c^+^ B cells. We therefore began by characterizing the systemic and tissue-level features of the model before focusing on the cellular and functional contribution of DN CD11c^+^ B cells.

**Figure 1:**
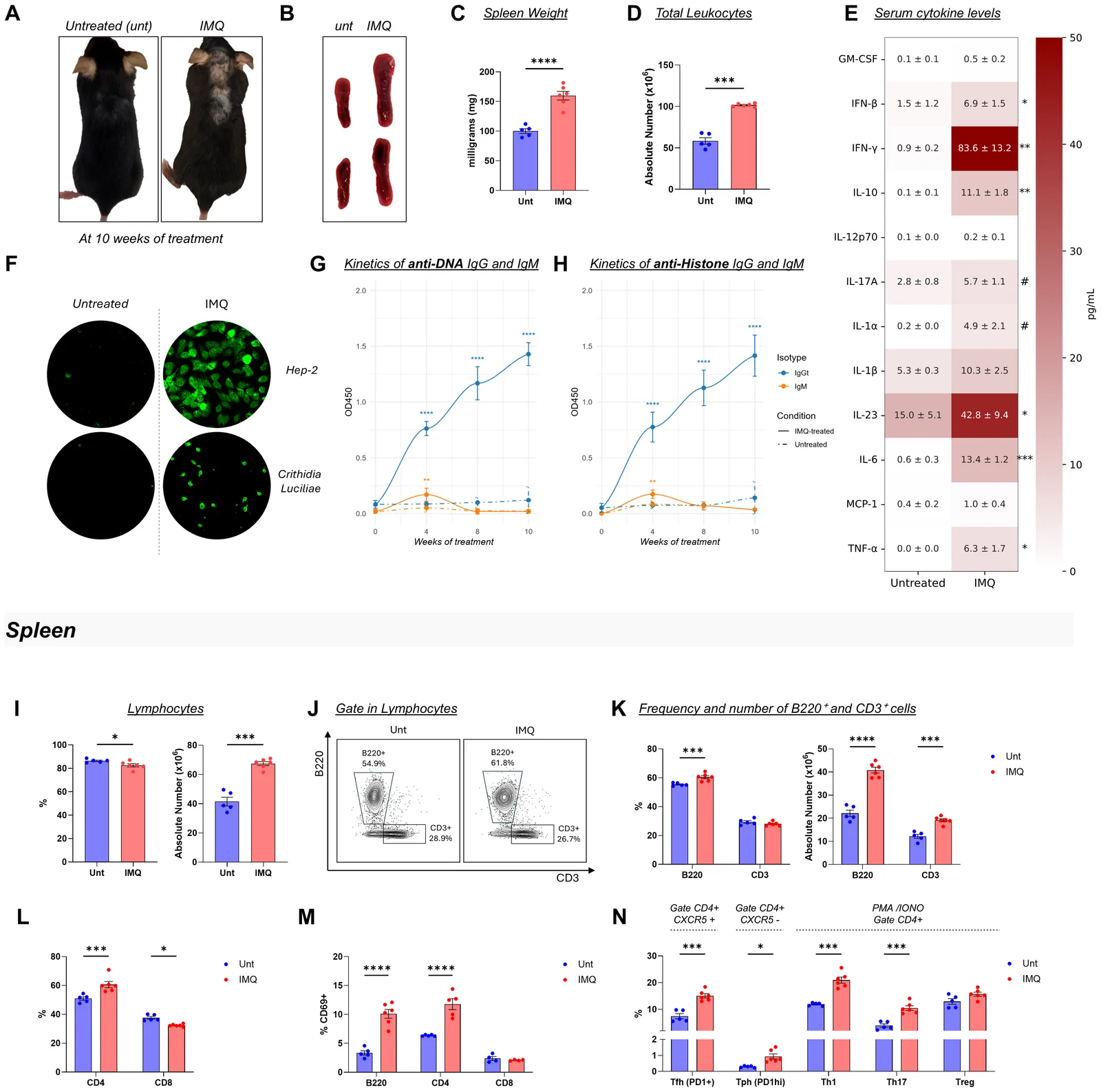
Chronic topical TLR7 stimulation induces systemic disease, splenomegaly, lymphocyte activation, and IgG-dominant autoantibody production. **(A)** Representative appearance of untreated (Unt; blue, n = 5) and IMQ-treated mice (red, n = 6) after 10 weeks of treatment, showing ruffled coat, alopecia, and reduced body condition in the IMQ group. **(B)** Representative spleens. **(C, D)** Quantification of spleen weight and total splenocyte numbers. **(E)** Serum cytokine concentrations (pg/mL) measured by LEGENDplex. **(F)** ANA reactivity on HEp-2 cells and anti-dsDNA reactivity on *Crithidia luciliae*, shown as positive/negative staining. **(G, H)** Kinetics of anti-dsDNA and anti-histone IgM and IgG autoantibodies throughout the 10-week treatment period, measured by ELISA as OD_450_ values (sera diluted 1:5000 for anti-dsDNA and anti-histone). **(I)** Percentage and absolute number of splenic lymphocytes. **(J)** Representative gating strategy within the lymphocyte compartment. **(K)** Frequencies and absolute numbers of B220^+^ B cells and CD3^+^ T cells. **(L)** Frequencies of CD4^+^ and CD8^+^ T cells. **(M)** CD69 expression on B220^+^ B cells and CD4^+^/CD8^+^ T cells (ex vivo). **(N)** CD4^+^ T helper subsets: Th1 and Th17 identified after PMA/ionomycin stimulation, while Tfh (CXCR5^+^ PD-1^+^), Tph (CXCR5^–^ PD-1^hi^), and Treg cells were analyzed ex vivo. Bars show mean ± SEM. Two-group comparisons were performed using Mann–Whitney tests; the serum cytokine panel **(E)** was analyzed with Benjamini–Hochberg FDR correction across analytes, and autoantibody kinetics **(G, H)** by linear mixed-effects models (treatment × time interaction p < 0.0001 for IgG) with emmeans-based timepoint contrasts; *p < 0.05, **p < 0.01, ***p < 0.001, ****p < 0.0001. For IL-17 and IL-1*α*, # indicates a statistical trend (0.05 < p ≤ 0.07).

After 10 weeks of topical IMQ administration on the ear, treated mice developed clear signs of systemic disease, including ruffled coat, weight loss, and areas of alopecia, which were absent in untreated controls (**Figure 1A**). Consistently, spleens from IMQ-treated mice were markedly enlarged, showing a significant increase in both weight and total cellularity compared with untreated controls (**Figures 1B–D**). Serum cytokine analysis revealed a broad proinflammatory profile in IMQ-treated mice, with significantly increased levels of IFN-*β*, IFN-*γ*, IL-6, TNF-*α*, IL-1*β*, IL-23 and IL-10 compared with controls (**Figure 1E**). IL-1*α* and IL-17 showed a nonsignificant trend toward higher concentrations, while GM-CSF, IL-12p70, and MCP-1 remained comparable to levels observed in untreated mice (**Figure 1E**). At the serological level, a slight and transient rise in IgM autoantibodies was observed after 4 weeks of treatment. By week 10, class-switched IgG autoantibodies were detectable by three orthogonal assays: HEp-2/Crithidia indirect immunofluorescence and dsDNA/histone ELISAs (**Figures 1F–H**; see also **Supplementary Figure 2A** for the longitudinal experimental design, **Supplementary Figure 2B** for autoantibody titers, and **Supplementary Figures 2C, D** for IgG subclass distribution).

Flow cytometric analysis showed a slight reduction in the overall percentage of splenic lymphocytes in IMQ-treated spleens, yet their absolute numbers were markedly increased compared with controls, reflecting the strong splenomegaly observed (**Figure 1I**; gating strategy shown in **Figure 1J**). Within this compartment, B220^+^ B cells were significantly expanded both in frequency and absolute numbers, while CD3^+^ T cells displayed a modest decrease in frequency but a clear increase in total numbers (**Figure 1K**). Further analysis revealed that CD4^+^ T cells were enriched, whereas CD8^+^ T cells showed a minor, but significant, reduction in frequency (**Figure 1L**). Assessment of the activation marker CD69 demonstrated a robust upregulation on B cells and CD4^+^ T cells, while no increase was detected on CD8^+^ T cells (**Figure 1M**). In line with this activation profile, CD4^+^ T helper subsets were skewed toward effector phenotypes: Th1, Th17, Tfh, and Tph cells were all significantly expanded in IMQ-treated mice, whereas Treg frequencies remained unchanged (**Figure 1N**). Together, our results confirm that chronic topical IMQ treatment drives systemic immune activation, splenomegaly, and the production of pathogenic autoantibodies. Consistent with previously established TLR7-driven lupus-like models (26), glycerin-treated controls did not develop splenomegaly or autoantibody elevations, and the systemic and serological features observed here (**Figures 1B–N**) recapitulate hallmark characteristics of TLR7-mediated autoimmunity.

### 3.2 Chronic TLR7 agonist treatment is associated with expansion of DN CD11c^+^ B cells (ABC-like cells) with an activated phenotype

**Figure 2:**
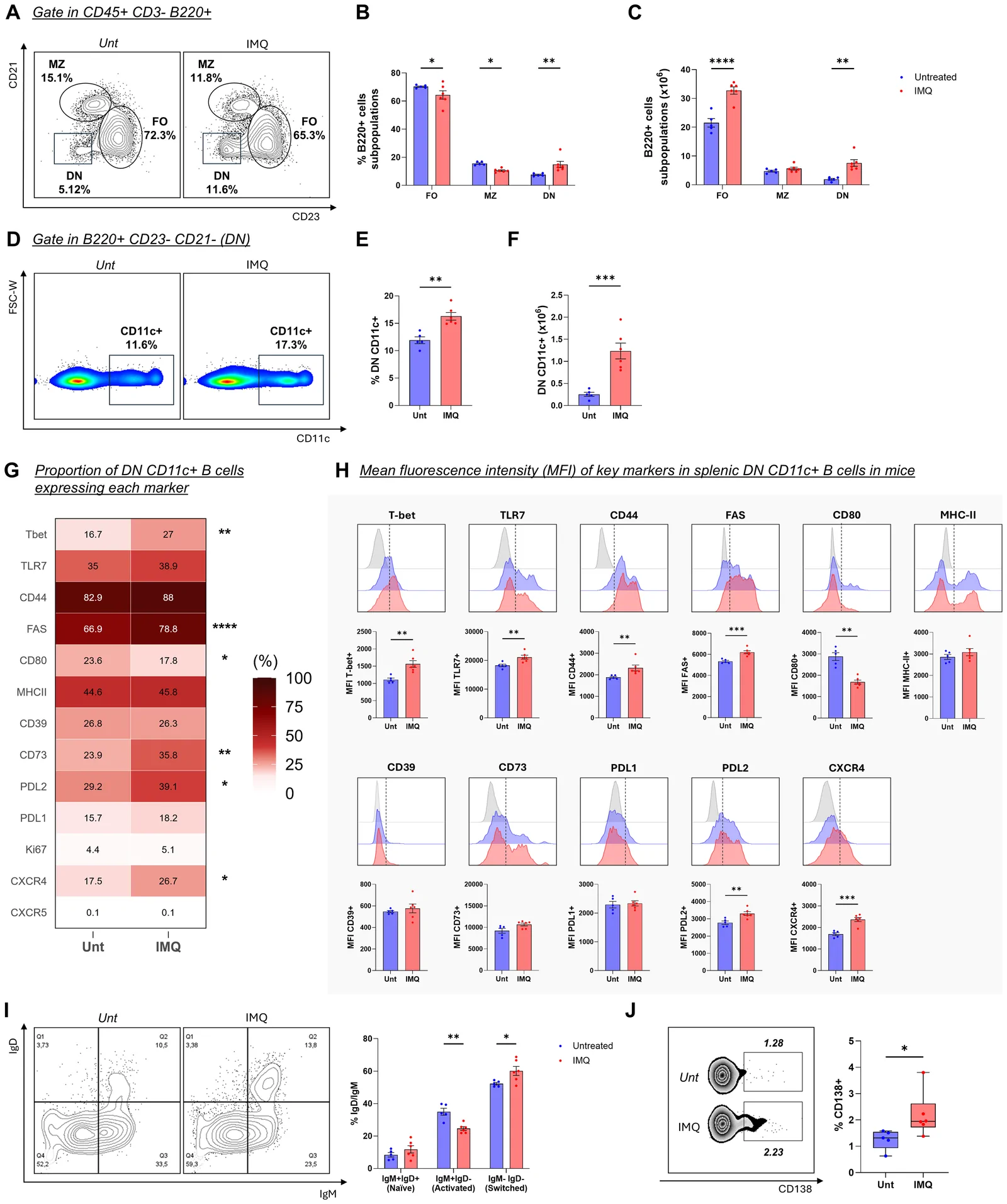
Chronic TLR7 stimulation induces expansion and phenotypic characterization of splenic DN CD11c^+^ B cells. **(A)** Gating strategy for splenic B cell subsets—follicular (FO), marginal zone (MZ), and double-negative (DN)—within CD45^+^CD3^–^B220^+^ lymphocytes in untreated (Unt; n = 5, blue) and IMQ-treated mice (n = 6, red). **(B, C)** Frequencies **(B)** and absolute numbers **(C)** of FO, MZ, and DN B cells. **(D)** Gating strategy for DN CD11c^+^ B cells (B220^+^CD21^–^CD23^–^CD11c^+^). **(E, F)** Frequencies **(E)** and absolute numbers **(F)** of DN CD11c^+^ B cells. **(G)** Proportion of DN CD11c^+^ B cells expressing the indicated phenotypic markers. **(H)** Mean fluorescence intensity (MFI) of the same markers. **(I)** Surface IgD and IgM expression profile of DN CD11c^+^ B cells, distinguishing naïve-like (IgD^+^IgM^+^), activated (IgD^–^IgM^+^), and class-switched (IgD^–^IgM^–^) subsets. **(J)** Frequencies of CD138^+^ DN CD11c^+^ B cells. Bars represent mean ± SEM. Two-group comparisons were performed using Mann–Whitney tests; the phenotypic marker panels **(G, H)** were analyzed with Benjamini–Hochberg FDR correction across markers. *p < 0.05, **p < 0.01, ***p < 0.001, ****p < 0.0001.

Analysis of splenic B cell subsets revealed IMQ-dependent alterations (**Figure 2**; see **Supplementary Figure 1A** for the splenic gating strategy). In IMQ-treated mice, the frequency of follicular (FO) and Marginal Zone (MZ) B cells was significantly reduced, whereas CD21^–^CD23^–^ double-negative (DN) B cells were markedly increased (**Figures 2A, B**). In absolute numbers, both FO and DN B cells were increased compared to controls, and DN CD11c^+^ B cells were significantly expanded in both frequency and absolute count (**Figures 2C, D–F**). Further phenotypic characterization of splenic DN CD11c^+^ B cells revealed increased frequencies of T-bet^+^, FAS^+^, CD73^+^, PD-L2^+^, and CXCR4^+^ cells, while the proportion of CD80^+^ DN CD11c^+^ B cells was reduced. The frequencies of TLR7^+^, CD44^+^, MHC-II, CD39^+^, PD-L1^+^., and Ki67^+^ cells remained comparable between groups (**Figure 2G**). As expected, DN CD11c^+^ B cells lacked CXCR5 expression, consistent with their extrafollicular phenotype (**Figure 2G**). Analysis of expression levels showed that DN CD11c^+^ B cells from IMQ-treated mice displayed significantly higher MFI for T-bet, TLR7, CD44, FAS, PD-L2, and CXCR4, whereas MFI values for MHC-II, CD39, CD73, and PD-L1 were not significantly different (**Figure 2H**). Notably, CD80 expression level decreased both in frequency and MFI (**Figures 2G, H**).

The analysis of surface IgD and IgM expression on DN CD11c^+^ B cells indicated that the majority of DN CD11c^+^ B cells in both untreated and IMQ-treated mice were class-switched (IgD^–^IgM^–^), with smaller proportions of naïve-like (IgD^+^IgM^+^) and activated (IgD^–^IgM^+^) cells (**Figure 2I**). However, IMQ-treated mice displayed a higher frequency of class-switched DN CD11c^+^ B cells and a concomitant reduction in the proportion of activated (IgD^–^IgM^+^) DN CD11c^+^ B cells compared to untreated controls, suggesting an enhanced progression toward a more differentiated phenotype upon chronic TLR7 stimulation (**Figure 2I**). In addition, although present at low frequencies, the proportions of CD138^+^ DN CD11c^+^ B cells were increased in the spleens of IMQ-treated mice (**Figure 2J**) when compared to untreated mice. Taken together, these data indicate that IMQ treatment is associated with the expansion of splenic DN CD11c^+^ B cell-like cells with an activated phenotype and enhanced class-switched and plasma cell differentiation.

### 3.3 Chronic TLR7 agonist treatment is associated with renal immune infiltration, including activated CD11c^+^ B cells

**Figure 3:**
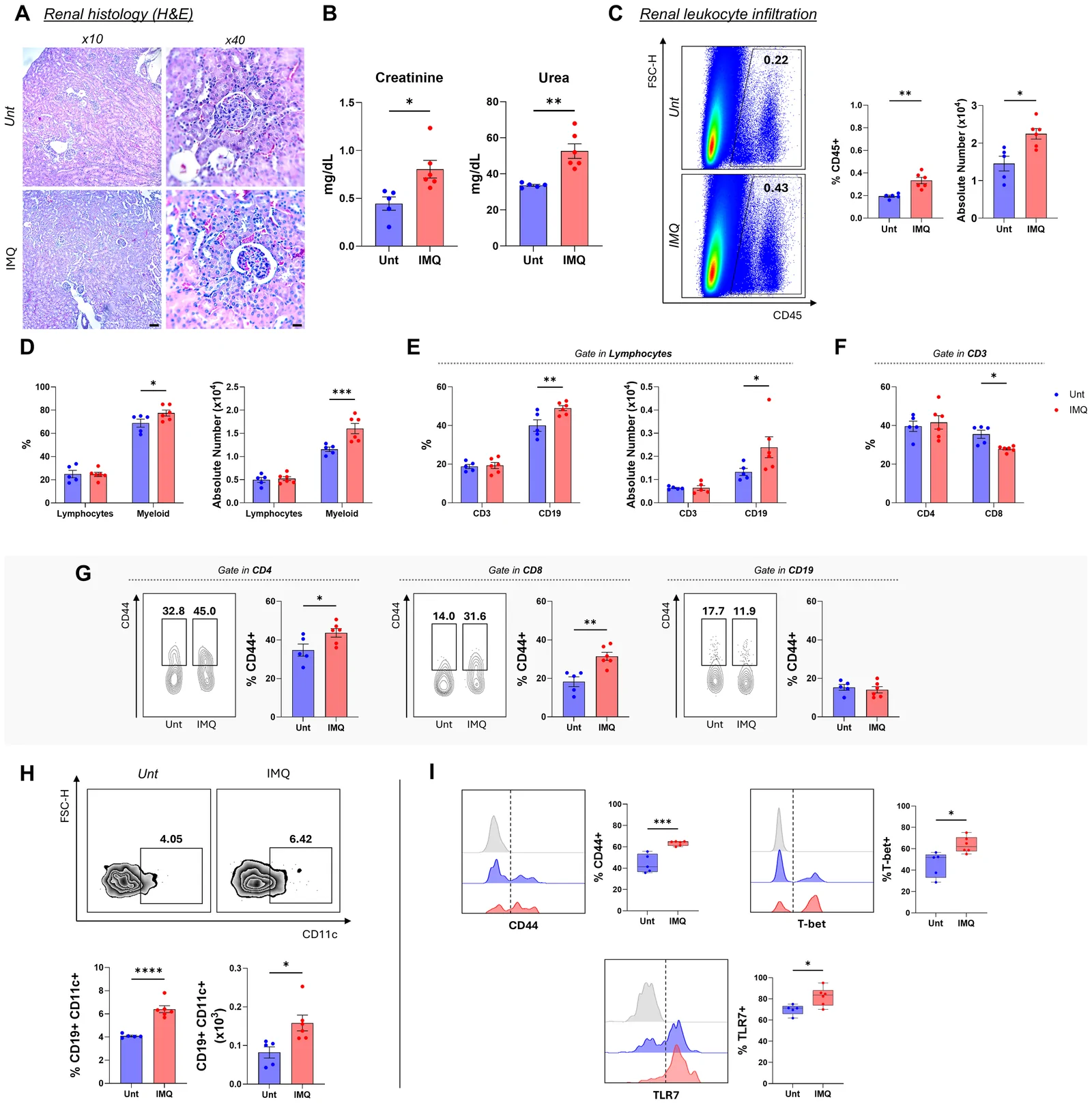
Renal alterations and immune cell infiltration in mice subjected to chronic TLR7 stimulation. **(A)** Representative kidney histology (H&E) from untreated (Unt; n = 5, blue) and IMQ-treated mice (n = 6, red) at 10× and 40× magnification (scale bars, 200 *μ*m and 20 *μ*m, respectively). **(B)** Serum creatinine and urea levels. **(C)** Gating strategy for renal CD45^+^ leukocytes and corresponding frequencies and absolute numbers. **(D)** Frequencies and absolute numbers of renal lymphoid and myeloid populations. **(E)** Frequencies and absolute numbers of renal CD3^+^ T cells and CD19^+^ B cells. **(F)** Frequencies of CD4^+^ and CD8^+^ T cells within renal CD3^+^ cells. **(G)** CD44 expression on renal CD4^+^ T cells, CD8^+^ T cells, and CD19^+^ B cells. **(H)** Gating strategy for renal CD11c^+^ B cells and their frequencies and absolute numbers. **(I)** CD44, T-bet, and TLR7 expression on renal CD11c^+^ B cells. Bars show mean ± SEM. Statistical analyses were performed using nonparametric tests (Mann–Whitney); the multiparameter marker panels **(G, I)** were analyzed with Benjamini–Hochberg FDR correction across markers. *p < 0.05, **p < 0.01, ***p < 0.001, ****p < 0.0001.

Given the presence of high titers of anti-dsDNA, ANA autoantibodies, systemic inflammatory features and the expansion of activated DN CD11c^+^ B cells in IMQ-treated mice, we next evaluated whether chronic TLR7 stimulation was associated with renal alterations. To this end, we assessed kidney histopathology, serum markers of renal function, and immune cell infiltration. Histological examination of H&E-stained kidney sections revealed only mild structural changes in IMQ-treated mice compared to untreated controls, characterized by subtle glomerular alterations and focal leukocyte accumulations, without overt tissue damage (**Figure 3A**). Despite the modest histopathological changes, serum creatinine and urea levels were significantly increased in IMQ-treated mice relative to controls (**Figure 3B**), suggesting functional alterations consistent with renal involvement, although hypercatabolism associated with systemic disease may also contribute to elevated urea levels. These functional changes may precede more pronounced structural injury.

Flow cytometric analysis of kidney cell suspensions revealed a significant increase in both the frequency and absolute number of CD45^+^ leukocytes infiltrating the kidneys of IMQ-treated mice (**Figure 3C**; see **Supplementary Figure 1B** for the renal gating strategy). The infiltrating compartment was enriched in myeloid cells, which were elevated in both relative and absolute terms (**Figure 3D**). We also detected a significant increase in renal B cells, observed at both the percentage and absolute number levels (**Figure 3E**). In contrast, the total frequencies of CD3^+^ T cells and CD4^+^ T cells remained unchanged, whereas the proportion of CD8^+^ T cells was reduced in IMQ-treated mice (**Figure 3F**). Notably, assessment of the activation marker CD44 showed significantly higher expression on CD4^+^ T cells, CD8^+^ T cells, but not on B cells from IMQ-treated mice, indicating a locally activated lymphocyte phenotype (**Figure 3G**). Importantly, a significant accumulation of CD11c^+^ B cells, consistent with the splenic DN CD11c^+^ B cell population identified above, was detected within the kidney (**Figure 3H**). As noted in Methods, mice were perfused via cardiac puncture with cold PBS to minimize intravascular contamination. These renal CD11c^+^ B cells displayed elevated expression of CD44, T-bet, and TLR7 (**Figure 3I**), consistent with an activated and pro-inflammatory profile.

Together, these findings demonstrate that chronic TLR7 stimulation drives immune cell recruitment to the kidney and promotes the local accumulation and activation of CD11c^+^ B cells. In the absence of urine protein measurements and glomerular IgG/C3 immunofluorescence, the renal changes reported here are interpreted as early renal immune activation rather than established lupus nephritis. Although IMQ-treated mice exhibited mild histological changes and increased renal immune cell infiltration, confirmation of lupus nephritis would require demonstration of proteinuria and glomerular immune-complex deposition.

### 3.4 DN CD11c^+^ B cells from imiquimod treated mice are highly responsive to TLR7 stimulation and are an important ex vivo source of autoreactive antibodies

**Figure 4:**
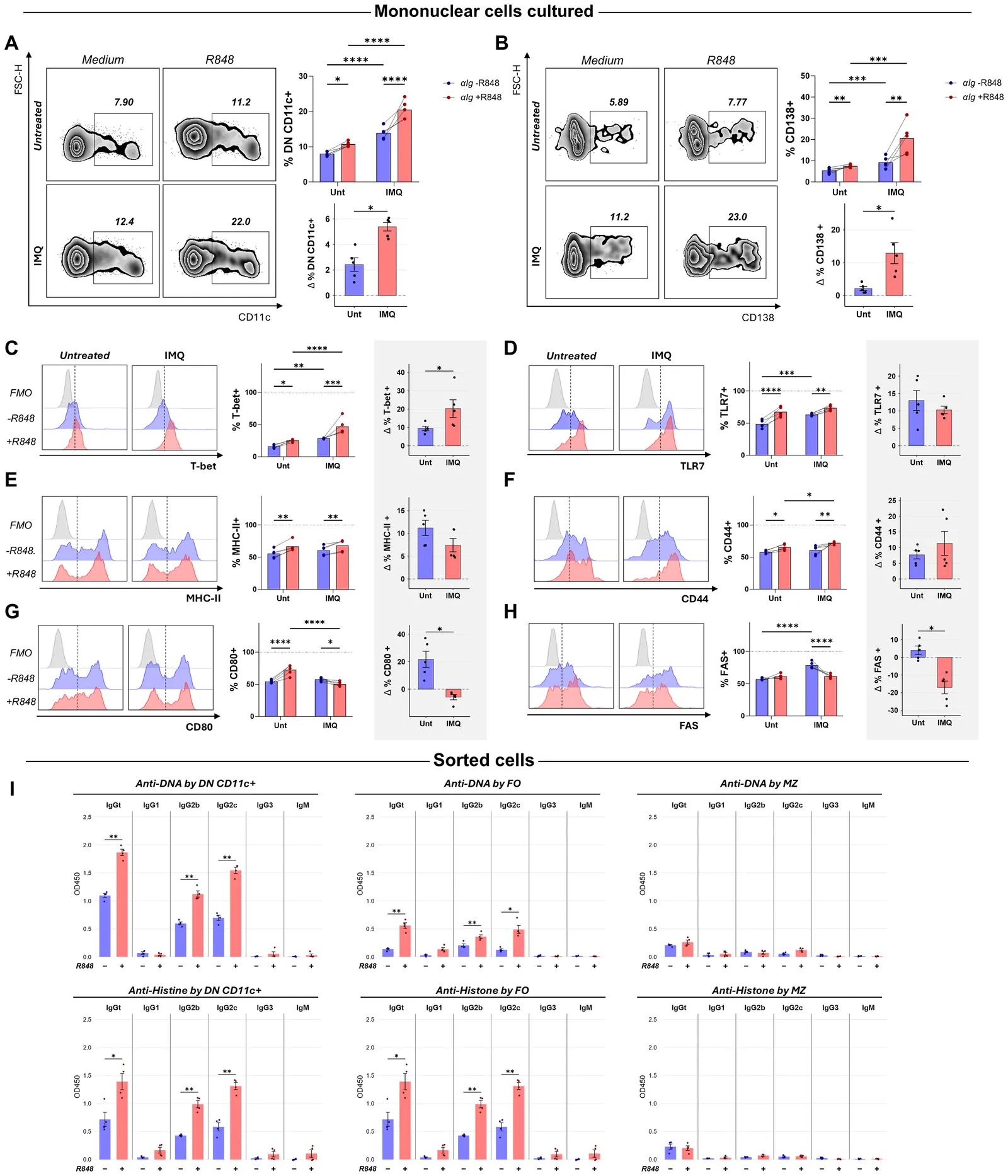
Ex vivo stimulation of splenic mononuclear cells and autoantibody production by sorted B cell subsets. **(A)** CD11c expression on DN CD11c^+^ B cells (B220^+^CD23^–^CD21^–^CD11c^+^) cultured for 24 h with anti-IgM/IgG (*α*Ig) alone or with *α*Ig plus R848, and corresponding frequencies and Δ% values (Δ = with R848 – without R848), in untreated (Unt; n = 5, blue) and IMQ-treated mice (n = 5, red). **(B)** CD138 expression under the same stimulation conditions, with frequencies and Δ% values. **(C–H)** Expression of T-bet **(C)**, TLR7 **(D)**, MHC-II **(E)**, CD44 **(F)**, CD80 **(G)**, and FAS **(H)** on DN CD11c^+^ B cells cultured with or without R848. Panels show representative histograms (FMO control, –R848, +R848), frequencies, and Δ% values. **(I)** Autoantibody production by sorted B cell subsets—DN CD11c^+^ B cells, follicular (FO), and marginal zone (MZ) B cells (DN CD11c^–^ B cells shown in **Supplementary Figure 2E**)—cultured for 72 h with *α*Ig alone or *α*Ig + R848. Shown are anti-dsDNA and anti-histone antibody levels across IgG total (IgGt), IgG1, IgG2b, IgG2c, IgG3, and IgM isotypes, measured in supernatants at a 1:2 dilution and read at 450 nm. Bars represent mean ± SEM. Stimulation experiments **(A–H**) were analyzed by two-way repeated-measures ANOVA (*in vivo* treatment × ex vivo R848 stimulation; individual mouse as a blocking factor), whereas Δ% comparisons and the sorted-cell autoantibody measurements **(I)** were assessed using Mann–Whitney tests. *p < 0.05, **p < 0.01, ***p < 0.001, ****p < 0.0001.

To evaluate how chronic TLR7 stimulation shapes the functional responsiveness of DN CD11c^+^ B cells, we cultured splenic mononuclear cells from untreated and IMQ-treated mice and stimulated them ex vivo with anti-IgM/IgG together with the TLR7 agonist R848 for 24 h. Stimulation increased the frequency of DN CD11c^+^ B cells in both groups, but the magnitude of this expansion (Δ%) was significantly greater in cells derived from IMQ-treated mice (**Figure 4A**). A similar pattern was observed for the antibody-secreting cell marker CD138: although both groups increased the proportion of CD138^+^ DN CD11c^+^ B cells upon stimulation, the rise was markedly higher in IMQ-derived cultures, consistent with an enrichment of effector-like DN CD11c^+^ B cells following chronic *in vivo* IMQ exposure (**Figure 4B**). T-bet expression also increased in DN CD11c^+^ B cells from both groups, with IMQ-treated mice displaying a significantly greater Δ% response (**Figure 4C**). In contrast, TLR7, MHC-II and CD44 expression increased to a comparable extent in DN CD11c^+^ B cells from both groups (**Figures 4D–F**). Interestingly, co-stimulatory and survival-associated molecules showed divergent patterns depending on prior *in vivo* treatment: stimulation increased CD80^+^ DN CD11c^+^ B cells only in untreated cultures, whereas CD80^+^ and FAS^+^ frequencies decreased in DN CD11c^+^ B cells from IMQ-treated mice (**Figures 4G, H**). Taken together, these ex vivo stimulation assays indicate that chronic TLR7 exposure not only expands DN CD11c^+^ B cells *in vivo* but also primes them for heightened activation and differentiation responses upon subsequent BCR and TLR7 engagement.

To determine whether this enhanced responsiveness translated into increased autoreactive effector function, we next assessed the capacity of sorted B cell subsets to produce autoantibodies. We purified splenic follicular (FO), marginal zone (MZ), double-negative (DN) B cells and DN CD11c^+^ B cells from IMQ-treated mice and cultured them for 72 h with anti-IgM/IgG, with or without R848. DN CD11c^+^ B cells displayed robust spontaneous autoantibody secretion upon BCR stimulation alone, producing high levels of anti-dsDNA and anti-histone IgG—particularly IgG2b and IgG2c (**Figure 4I**). Co-stimulation through TLR7 (anti-IgM/IgG + R848) further amplified these outputs, demonstrating a strong synergistic effect of BCR and TLR7 signaling in driving DN CD11c^+^ B cell effector function. FO B cells produced only low amounts of autoantibodies under the same conditions, whereas MZ B cells failed to generate detectable autoreactive IgG (**Figure 4I**); sort-purified DN CD11c^–^ B cells likewise produced negligible autoantibodies under the same conditions (**Supplementary Figure 2E**). These results identify DN CD11c^+^ B cells as an important ex vivo source of anti-dsDNA and anti-histone IgG; however, the magnitude of this effect may be influenced by the donor context and effector subset composition.

### 3.5 Pharmacological engagement of ADORA2A restrains DN CD11c^+^ B cell expansion and autoreactive IgG production in chronic TLR7-driven autoimmunity

**Figure 5:**
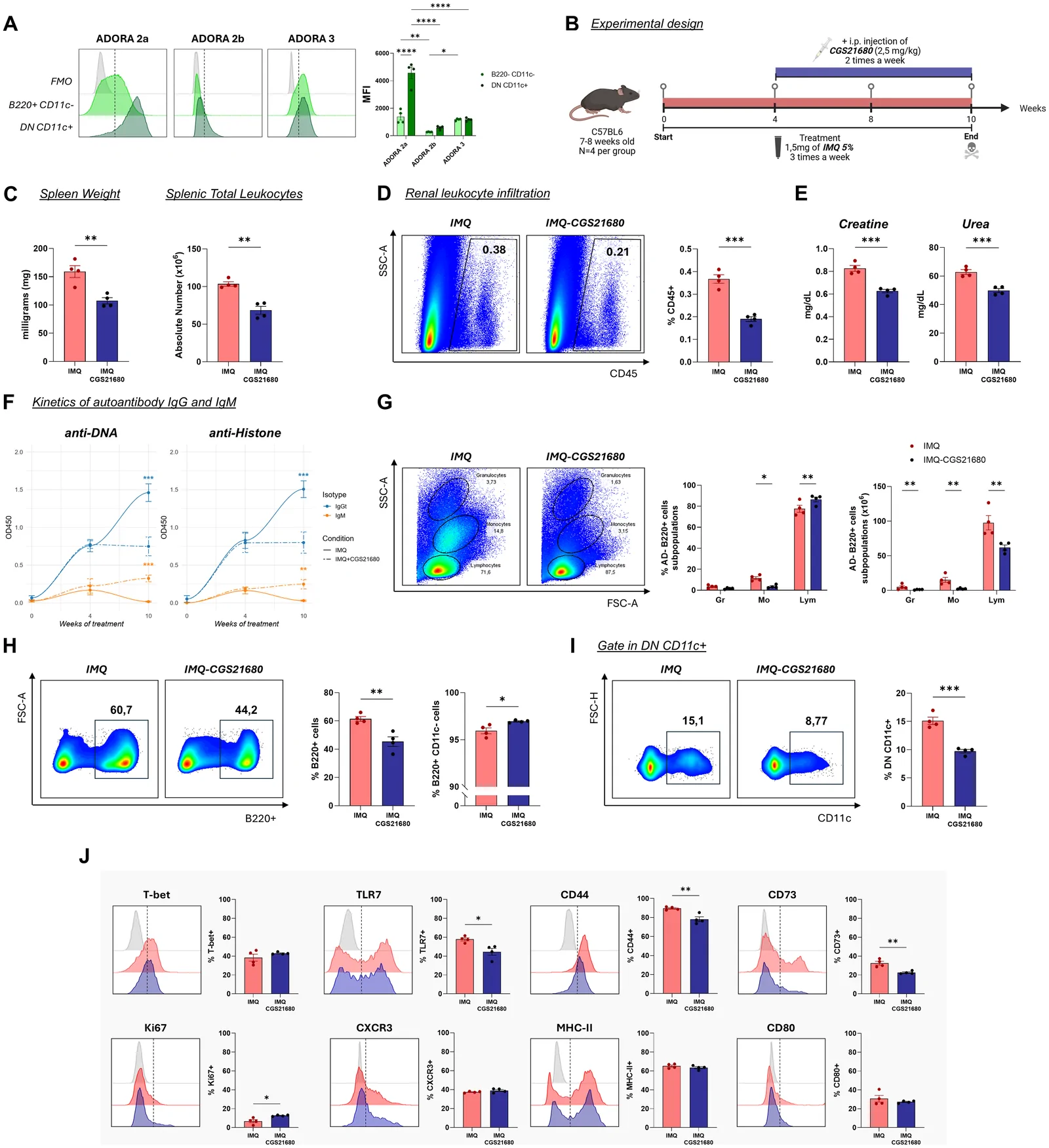
Pharmacological activation of ADORA2A during chronic TLR7 stimulation. **(A)** Expression of ADORA2A, ADORA2B, and ADORA3 on splenic B220^+^CD11c^–^ B cells and DN CD11c^+^ B cells, shown with representative histograms and quantification. **(B)** Schematic of the experimental design: both groups received topical IMQ for 10 weeks; the IMQ+CGS-21680 group additionally received CGS-21680 (2.5 mg/kg, i.p.) twice per week starting at week 4. n = 4 mice per group. **(C)** Spleen weight and total splenic leukocyte numbers at week 10. **(D)** Flow cytometry gating of renal CD45^+^ leukocytes and their frequencies. **(E)** Serum creatinine and urea levels. **(F)** Kinetics of anti-dsDNA and anti-histone IgM and IgG autoantibody levels over the 10-week treatment period, measured by ELISA and expressed as OD_450_ values (sera diluted 1:5000). **(G)** Frequencies and absolute numbers of splenic myeloid, granulocyte, and lymphocyte subsets. **(H)** Frequencies and absolute numbers of splenic B220^+^ B cells and B220^+^CD11c^–^ B cells. **(I)** Gating strategy for splenic DN CD11c^+^ B cells (B220^+^CD21^–^CD23^–^CD11c^+^) and corresponding frequencies and absolute numbers. **(J)** Expression of T-bet, TLR7, CD44, CD73, Ki67, CXCR3, MHC-II, and CD80 in splenic DN CD11c^+^ B cells, shown with representative histograms and quantification. Bars represent mean ± SEM. Comparisons were performed using unpaired two-tailed Student’s t tests (with Welch’s correction; n = 4 per group); autoantibody kinetics **(F)** were analyzed by linear mixed-effects models (treatment × time interaction p < 0.0001), and the multiparameter phenotyping panel **(J)** with Benjamini–Hochberg FDR correction. *p < 0.05, **p < 0.01, ***p < 0.001, ****p < 0.0001.

Given that DN CD11c^+^ B cells were identified as an important source of autoreactive antibodies, we next evaluated whether selective targeting of this subset could mitigate systemic autoimmunity. Previous reports indicate that DN CD11c^+^ B cells exhibit high expression of adenosine receptors, particularly the A2A receptor (ADORA2A). Consistent with this, DN CD11c^+^ B cells expressed markedly higher levels of ADORA2A compared with CD11c^–^ B cells, whereas ADORA2B and ADORA3 were expressed at lower levels in both populations (**Figure 5A**).

Based on these findings, we designed a therapeutic intervention in which one group of mice received IMQ for 10 weeks (IMQ group), while a second group received IMQ and began treatment with the A2A receptor agonist CGS-21680 starting at week 4 (IMQ+CGS group; experimental design in **Figure 5B**). At week 10, CGS-21680-treated mice displayed significantly reduced spleen weight and total splenic cellularity compared with IMQ controls (**Figure 5C**). Renal immune infiltration was also diminished in CGS-21680-treated animals, as evidenced by a significant reduction in CD45^+^ leukocytes within the kidney (**Figure 5D**). In addition, serum urea and creatinine were reduced in the IMQ+CGS-21680 group (**Figure 5E**).

Longitudinal measurement of autoantibodies revealed distinct kinetics for IgM and IgG. IgM anti-dsDNA and anti-histone levels rose comparably in both groups by week 4 and thereafter diverged, declining in IMQ-treated mice yet remaining significantly elevated in the IMQ+CGS-21680 group. In contrast, the marked increase in IgG autoantibodies observed in IMQ-treated mice was prevented in the IMQ+CGS-21680 group, in which IgG titers remained at week 4 levels (**Figure 5F**; the corresponding IgG subclass responses are shown in **Supplementary Figures 3A, B**). *In vivo* A2A activation thus reduced DN CD11c^+^ B cell frequencies and significantly decreased circulating autoreactive IgG, consistent with a functional contribution of DN CD11c^+^ B cells to the antibody pool.

Flow cytometric analysis showed that the overall composition of splenic myeloid, granulocyte, and lymphocyte subsets was largely preserved in CGS-21680-treated mice (**Figure 5G**). Within the B cell compartment, CGS-21680 treatment decreased the overall frequency of B220^+^ B cells but increased the proportion of B220^+^CD11c^–^ conventional B cells relative to IMQ controls (**Figure 5H**). Importantly, DN CD11c^+^ B cells were markedly reduced in both frequency and absolute number in CGS-treated mice (**Figure 5I**). Phenotypic analysis of residual DN CD11c^+^ B cells revealed lower frequencies of TLR7^+^, CD44^+^, and CD73^+^ cells following CGS-21680 treatment, whereas expression levels of T-bet, CXCR3, MHC-II, and CD80 were not significantly altered (**Figure 5J**). Together, these data demonstrate that pharmacological activation of the A2A pathway is associated with reduced DN CD11c^+^ B cell accumulation, diminished effector activation, and lower autoreactive IgG levels in this IMQ-induced lupus-like model. Definitive attribution of these effects to ADORA2A signaling will require antagonist reversal, dose–response testing, and genetic loss-of-function approaches.

## 4 Discussion

Chronic activation of TLR7 is sufficient to drive not only the expansion but also the functional programming of DN CD11c^+^ B cells (an ABC-like population) in a lupus-like model induced by IMQ. Here, we identify DN CD11c^+^ B cells as a major autoreactive B cell subset capable of producing class-switched autoantibodies and infiltrating the kidney at early stages of disease. In addition, we show that adenosine A2A receptor signaling is associated with restrained DN CD11c^+^ B cell expansion and effector function, highlighting a potential strategy to modulate early autoimmune responses.

A central finding of this study is that sustained innate immune activation, in the absence of genetic predisposition, is sufficient to induce both the expansion and differentiation of DN CD11c^+^ B cells toward a pathogenic phenotype. While DN CD11c^+^ B cell accumulation has been widely described in aging and spontaneous lupus models, our results extend these observations by showing that chronic TLR7 engagement alone can promote remodeling of the B cell compartment toward non-canonical and autoreactive states (6). This shift is consistent with increasing evidence supporting a role for extrafollicular pathways in the generation of pathogenic humoral responses in systemic lupus erythematosus (SLE) (31, 36). Extrafollicular (EF) B cell responses lack the stringent checkpoints found in germinal centers, making them less controlled; while EF pathways are highly effective at producing rapid, early antibodies during infections, they also represent a primary driver of pathogenic autoantibodies in autoimmunity (48). The ABC compartment is increasingly recognized as a heterogeneous and dynamic population that may arise from distinct developmental pathways, including extrafollicular and follicular responses (32). The CXCR5-negative, T-bet+ phenotype of the cells described here is consistent with an association with extrafollicular responses, but our data do not establish their developmental origin, which may be heterogeneous. Importantly, our data indicate that DN CD11c^+^ B cells are not merely expanded but exhibit features consistent with functional priming. DN CD11c^+^ B cells from IMQ-treated mice display enhanced responsiveness to TLR7 stimulation and preferentially upregulate CD11c, T-bet, and CD138, supporting a phenotype associated with plasmablast-like differentiation (18, 19). In contrast, other activation markers such as MHC-II, CD44, and TLR7 itself do not show differential inducibility, suggesting that chronic *in vivo* stimulation may selectively reinforce specific effector programs rather than globally amplifying activation capacity. This phenotype is compatible with a state of partial pre-activation or “poised differentiation” described in chronic inflammatory settings (14).

These changes occur within a systemic inflammatory environment characterized by increased levels of both type I and type II interferons. The concurrent elevation of IFN-*β* and IFN-*γ* suggests coordinated activation of innate and adaptive immune pathways. Although the cellular sources of these cytokines were not directly determined in our study, previous work using IFN-*γ* reporter mice has shown that, in IMQ-induced lupus, CD4^+^ effector T cells—including follicular helper T (Tfh) cells—are the major producers of TLR7-driven IFN-*γ* (39). Consistent with this and with the broader role of IFN-*γ* in TLR7-mediated autoimmunity, we observed an expansion of Th1 cells in IMQ-treated animals. Notably, our study extends prior work by providing a broader characterization of CD4^+^ T cell heterogeneity in this model, revealing increases not only in Th1 and Th17 cells, but also in T follicular helper (Tfh) and peripheral helper T (Tph) cells. The expansion of Tph cells is particularly noteworthy given the emerging role of this subset in supporting B cell responses and autoantibody production in autoimmune diseases, including SLE (49–51). To our knowledge, the contribution of Tph cells has not been previously examined in the IMQ-induced lupus model. Given their established role in promoting extrafollicular B cell responses in lupus, their presence suggests a potential mechanistic link between chronic TLR7 activation, IFN-*γ* production, and the emergence of autoreactive B cell responses.

When isolated and stimulated under defined conditions, DN CD11c^+^ B cells produced high levels of anti-dsDNA and anti-histone IgG, whereas FO and MZ B cells showed minimal or no autoreactive output. These findings are consistent with previous studies identifying ABCs as major contributors to autoreactive IgG production in lupus (6, 52). This selective capacity, together with the predominance of class-switched IgG2c and IgG2b isotypes, supports the pathogenic potential of DN CD11c^+^ B cell-derived responses (18). The enrichment of CD138^+^ cells within the DN CD11c^+^ B cell compartment suggests that these cells may be predisposed toward differentiation into antibody-secreting effectors, although this feature may partially contribute to their increased ex vivo output and should be interpreted with caution. These cells do not represent a population competing with other expanded subsets, but rather the effector compartment upstream of the autoreactive antibody-secreting cells, since a plasmablast or plasma cell represents a terminal differentiation state rather than a parallel lineage, and CD138+ antibody-secreting cells are enriched within this compartment (**Figure 2J**). In our study, DN CD11c+ B cells are defined by CD11c expression within the CD21−CD23− gate, with T-bet, CXCR5 and IgD/IgM used as phenotypic descriptors. On these markers the population corresponds to the murine equivalent of the human ABC/DN2 subset (16). The human DN/DN2 framework is based on IgD and CD27; CD27, however, is not a reliable marker for defining murine memory or B cell subsets, which are instead characterized by CD80, PD-L2 and CD73 (53).

Beyond their systemic role, DN CD11c^+^ B cells were also detected within renal tissue, where they displayed an activated T-bet^+^TLR7^+^ phenotype (54). Their presence, together with increased leukocyte infiltration and early functional alterations, suggests that these cells may contribute to the initial phases of tissue-specific inflammation. However, given the relatively mild renal phenotype in this model, further studies will be required to determine the extent to which CD11c^+^ B cells directly contribute to tissue pathology.

A key translational aspect of this study is the identification of adenosine A2A receptor signaling as a regulator of DN CD11c^+^ B cell responses. DN CD11c^+^ B cells exhibited increased expression of ADORA2A, providing a rationale for therapeutic targeting (46). A2A receptor agonism has been evaluated in wild-type mice during infection and in genetically predisposed lupus models such as MRL/lpr and SLE123 (46), and A2A engagement protects against renal injury in lupus-prone mice (55). Our study extends this approach to an inducible, TLR7-driven model in non-autoimmune-prone mice, a setting in which it had not been tested, and describes distinct IgM and IgG autoantibody kinetics under treatment. A2A engagement acts B cell-intrinsically during infection (46), although in our systemic treatment setting we cannot separate direct from indirect effects. Pharmacological activation of this pathway using CGS-21680 was associated with reduced DN CD11c^+^ B cell accumulation, attenuation of their activated phenotype, and decreased production of class-switched autoantibodies. These changes were accompanied by improved renal parameters and reduced immune cell infiltration. Together with previous studies demonstrating that A2A receptor signaling restrains pathogenic T cell responses and germinal center reactions (56, 57), our findings support a broader immunoregulatory role for this pathway. However, as these experiments relied on systemic pharmacological modulation, the relative contribution of direct effects on DN CD11c^+^ B cells versus indirect effects on other immune or stromal populations cannot be determined (58–60).

Together, our results are consistent with a model in which chronic TLR7 signaling promotes a feed-forward loop linking innate immune activation, interferon production, CD4^+^ T cell polarization, and DN CD11c^+^ B cell differentiation through extrafollicular pathways. In this context, DN CD11c^+^ B cells emerge as key effector cells that bridge innate sensing and adaptive autoreactivity, contributing to the production of pathogenic autoantibodies and tissue-specific immune inflammation.

Several limitations of this study should be considered. First, although IMQ-treated mice exhibited renal immune infiltration, mild histological alterations, and functional changes, the absence of proteinuria assessment and glomerular IgG/C3 immunofluorescence precludes definitive evaluation of lupus nephritis. Second, because A2A receptor modulation was achieved through systemic administration of CGS-21680, the relative contribution of direct effects on DN CD11c^+^ B cells versus indirect effects on other immune populations remains to be determined. Finally, although elevated levels of IFN-*β* and IFN-*γ* were observed, the cellular sources of these cytokines were not directly identified. Our study provides associative and ex vivo functional evidence for a pathogenic role of these cells rather than a formal *in vivo* loss-of-function demonstration in this model. The causal contribution of CD11c+ B cells to lupus is established in other settings (11, 32), and a dedicated loss-of-function experiment within the inducible TLR7 model, such as conditional deletion of CD11c+ B cells or adoptive transfer of sorted subsets, represents an important direction for future work. We also did not assess T helper subsets, including Tfh and Tph cells, in the A2A-treated group, so the contribution of T cell-mediated effects to the reduction of DN CD11c+ B cells remains to be determined. Finally, an unbiased transcriptomic characterization would further resolve the molecular identity of these cells and their relationship to previously described ABC/DN2 subsets. Such analyses were not undertaken here and represent an important direction for future work. Future studies combining cell-specific genetic approaches and detailed characterization of renal pathology will help further define the mechanisms linking TLR7 signaling, DN CD11c^+^ B cell responses, and tissue inflammation.

In conclusion, our study identifies DN CD11c^+^ B cells (ABC-like cells) as a central effector population in TLR7-driven lupus-like autoimmunity, linking chronic innate immune activation to the generation of pathogenic humoral responses. We show that DN CD11c^+^ B cells are expanded and exhibit features consistent with functional priming, contributing to autoreactive antibody production and early tissue inflammation. Furthermore, our findings suggest that modulation of A2A receptor signaling may represent a strategy to restrain DN CD11c^+^ B cell responses. These results provide mechanistic insight into the pathways underlying autoimmune activation and highlight DN CD11c^+^ B cells as a potential therapeutic target in interferon- and TLR-driven autoimmune diseases.

## Data Availability

The raw data supporting the conclusions of this article will be made available by the authors, without undue reservation.

## Supplementary material

Supplementary Figure 1 Gating strategies for splenic **(A)** and kidney **(B)** immune cell subpopulations, and representative sort panel for splenic B cell subsets **(C)**. **(A)** Splenocytes were gated on singlets (FSC-H/FSC-A) and live cells (AD⁻; LIVE/DEAD™ Fixable Aqua), followed by selection of lymphocytes based on FSC-A/SSC-A. Lymphocytes were separated into B220⁺ B cells and CD3⁺ T cells. B220⁺ cells were subdivided into follicular (FO; CD23⁺ CD21^low/variable), marginal zone (MZ; CD23⁻ CD21⁺), and double-negative (DN; CD23⁻ CD21⁻) subsets. DN CD11c⁺ B cells were identified within the DN compartment as CD11c⁺ cells and further characterised for phenotypic and activation markers including T-bet, TLR7, CD44, FAS, CD39, CD73, CD138, CXCR5, among others. Representative FMO controls and histograms comparing untreated and IMQ-treated mice are shown. CD3⁺ T cells were subdivided into CD4⁺ and CD8⁺ populations and analysed for CD44 expression. Within the CD4⁺ compartment, regulatory T cells (FoxP3⁺), T follicular helper cells (Tfh; CXCR5⁺ PD-1⁺), and T peripheral helper cells (Tph; CXCR5⁻ PD-1hi) were identified. Following PMA/ionomycin stimulation, intracellular IFN-γ (Th1) and IL-17A (Th17) production was assessed. **(B)** Kidney single-cell suspensions were gated on singlets, followed by CD45⁺ leukocytes and live cells (AD⁻). CD45⁺ cells were separated into lymphoid and myeloid compartments based on FSC-A/SSC-A, and CD11b⁺ myeloid cells were identified. Lymphocytes were further subdivided into CD19⁺ B cells and CD3⁺ T cells. Renal CD11c⁺ B cells (within the CD19⁺ B cell compartment) were analysed for expression of T-bet, TLR7, and CD44 using FMO-guided gating and representative histograms from untreated and IMQ-treated mice. CD3⁺ T cells were separated into CD4⁺ and CD8⁺ subsets and evaluated for CD44 expression. **(C)** Representative dot plots showing the gating strategy used for FACS sorting of splenic B-cell subsets from pooled spleens of IMQ-treated mice. Pre-sort plots (top row) display the sequential gating from singlets, lymphocytes, AD⁻ live cells, and B220⁺ B cells, followed by CD23/CD21 separation into follicular (FO), marginal zone (MZ), and double-negative (DN) populations, and finally CD11c⁻/CD11c⁺ subdivision within the DN compartment. Post-sort plots (bottom row) confirm the purity (>97%) of each sorted population: FO, MZ, DN CD11c⁻, and DN CD11c⁺ B cells.

Supplementary Figure 2 Kinetics and subclass distribution of anti-DNA and anti-histone autoantibodies during IMQ-induced lupus. **(A)** Experimental design. C57BL/6 mice (7–8 weeks old; n = 5 per group) were assigned to two groups and treated topically for 10 weeks with either 5% imiquimod (IMQ; 1.5 mg, three times per week) or vehicle control (glycerin). Blood samples were collected by submandibular puncture at weeks 0, 4, 8 and 10. **(B)** Serum anti-DNA and anti-histone autoantibody titres, expressed as the maximum positive dilution (blank + 2 SD threshold), in vehicle-treated (Unt.) and IMQ-treated mice at the indicated time points. **(C, D)** IgG subclass distribution of serum anti-DNA **(C)** and anti-histone **(D)** autoantibodies over the course of IMQ treatment. ELISA absorbance was measured at a fixed dilution (1:5000) for IgG1, IgG2b, IgG2c and IgG3. **(E)** Autoantibody production by sorted DN CD11c⁻ B cells (B220⁺ CD23⁻ CD21⁻ CD11c⁻) after TLR7 stimulation. DN CD11c⁻ B cells were sorted from spleens of IMQ-treated mice and cultured for 72 h with anti-IgM/IgG alone (−) or anti-IgM/IgG plus R848 (+). Supernatants (1:2 dilution) were analysed by ELISA for anti-DNA (left) and anti-histone (right) autoantibodies across Ig isotypes (IgGt, IgG1, IgG2b, IgG2c, IgG3 and IgM) and read at 450 nm. Bars represent mean ± SEM. *p < 0.05; **p < 0.01; ***p < 0.001; ****p < 0.0001.

Supplementary Figure 3 A2A agonism modulates IgG subclass autoantibody responses. **(A, B)** Serum anti-DNA **(A)** and anti-histone **(B)** IgG subclass responses in IMQ-treated mice in the absence (IMQ) or presence of the A2A receptor agonist CGS-21680 (IMQ + CGS-21680). ELISA OD450 was measured at a fixed dilution (1:5000) for IgG1, IgG2b, IgG2c and IgG3 at weeks 0, 4 and 10 of treatment. Bars represent mean ± SEM, with individual mice shown as points. Statistical significance indicates comparisons between IMQ and IMQ + CGS-21680 within each time point and IgG subclass. *p < 0.05; **p < 0.01; ***p < 0.001; ****p < 0.0001.
