# Correction: Chronic TLR7 activation promotes expansion of activated CD11c+ double-negative B cells and autoreactive humoral responses in lupus-like autoimmunity

**DOI:** 10.3389/fimmu.2026.2002015

**Published:** 2026-10-02

**Authors:** Fernando N. Ferreyra, Laura Almada, Maria S. Martinez, Yair A. Chocobar, Rubén D. Motrich, Juan Pablo Mackern-Oberti, Adriana Gruppi, Virginia E. Rivero

**Affiliations:** 1 Federation of Clinical Immunology Societies (FOCIS) Center of Excellence, Centro de Inmunología Clínica de Córdoba (CICC), Córdoba, Argentina; 2 Centro de Investigaciones en Bioquímica Clínica e Inmunología (CIBICI)-Consejo Nacional de Investigaciones Científicas y Técnicas (CONICET), Facultad de Ciencias Químicas, Universidad Nacional de Córdoba, Córdoba, Argentina; 3 Instituto de Medicina y Biología Experimental de Cuyo (IMBECU), Centro Científico Tecnológico (CCT) Consejo Nacional de Investigaciones Científicas y Técnicas (CONICET), Mendoza, Argentina; 4 Universidad Nacional de Cuyo, Facultad de Ciencias Médicas, Mendoza, Argentina

**Keywords:** ABC-like, ADORA2A, autoantibodies, DN CD11c+ B cells, extrafollicular response, imiquimod, lupus, TLR7

The funder “National Institutes of Health (NIH) – 1R01AI116432-06” to Adriana Gruppi was erroneously omitted. The correct **Funding** statement is:

“The author(s) declared that financial support was received for this work and/or its publication. This work was supported by the Agencia Nacional de Promoción Científica y Tecnológica (ANPCyT-FONCyT), the Consejo Nacional de Investigaciones Científicas y Técnicas (CONICET), the Secretaría de Ciencia y Tecnología de la Universidad Nacional de Córdoba (SECyT-UNC), and the National Institutes of Health (NIH) - 1R01AI116432-06. The funders had no role in study design, data collection and analysis, decision to publish, or preparation of the manuscript.”

The original version of this article has been updated.

